# New Developments in T Cell Immunometabolism and Therapeutic Implications for Type 1 Diabetes

**DOI:** 10.3389/fendo.2022.914136

**Published:** 2022-06-10

**Authors:** Mengdi Zhang, Yanyan Zhou, Zhiguo Xie, Shuoming Luo, Zhiguang Zhou, Jiaqi Huang, Bin Zhao

**Affiliations:** ^1^National Clinical Research Center for Metabolic Diseases, Key Laboratory of Diabetes Immunology, Ministry of Education, and Department of Metabolism and Endocrinology, The Second Xiangya Hospital of Central South University, Changsha, China; ^2^Department of Critical Care Medicine, The Second Xiangya Hospital of Central South University, Changsha, China

**Keywords:** type 1 diabetes, T cell, T cell differentiation and function, T cell metabolism, autoimmunity

## Abstract

Type 1 diabetes (T1D) is an autoimmune disease mediated by T cells and is becoming a serious public health threat. Despite the increasing incidence rate of T1D worldwide, our understanding of why T1D develops and how T cells lose their self-tolerance in this process remain limited. Recent advances in immunometabolism have shown that cellular metabolism plays a fundamental role in shaping T cell responses. T cell activation and proliferation are supported by metabolic reprogramming to meet the increased energy and biomass demand, and deregulation in immune metabolism can lead to autoimmune disorders. Specific metabolic pathways and factors have been investigated to rectify known deficiencies in several autoimmune diseases, including T1D. Most therapeutic strategies have concentrated on aerobic glycolysis to limit T cell responses, whereas glycolysis is the main metabolic pathway for T cell activation and proliferation. The use of metabolic inhibitors, especially glycolysis inhibitors may largely leave T cell function intact but primarily target those autoreactive T cells with hyperactivated metabolism. In this review, we provide an overview of metabolic reprogramming used by T cells, summarize the recent findings of key metabolic pathways and regulators modulating T cell homeostasis, differentiation, and function in the context of T1D, and discuss the opportunities for metabolic intervention to be employed to suppress autoreactive T cells and limit the progression of β-cell destruction.

## Introduction

T1D is a chronic immune-metabolic disease and is becoming a serious public health threat (1). Over the past three decades, the incidence of T1D has escalated worldwide, afflicting as many as 10 million people (2, 3). The pathogenesis of T1D is complicated, and available data suggest that T1D arises due to the combination of genetically determined susceptibility, environmental factors, and impairment of immunity, which eventually leads to the breakdown of immune tolerance to self (4, 5). It was demonstrated that autoreactive CD4+ and CD8+ T cells that infiltrate the islets of T1D patients play a key role in the process of β-cell destruction (6, 7). Thus, those autoreactive T cells are regarded as a potential target for immune-based interventions aiming to combat T1D (8–11).

Recent advances in metabolomics, transgenic mice and immunometabolism have shown that metabolic adaptation plays a crucial role in shaping T cell responses (12–14). T cell activation is linked to metabolic reprogramming to meet the increased energy and biomass demand (15, 16). Binding of antigen to T cell receptor (TCR) initiates the activation of naïve T cells, which leads to a metabolic program shift from oxidative phosphorylation (OXPHOS) to robust aerobic glycolysis for rapid clonal proliferation and effector functions (17–19). In recent years, many exciting findings have uncovered novel metabolic pathways and key molecules that could be applied to improve the governance of autoimmunity and guide the treatment of autoimmune diseases (20). The use of metabolic inhibitors, especially glycolysis inhibitors, may largely leave T cell function intact but primarily target autoreactive T cells with hyperactivated metabolism (9, 20). This review aims to provide an overview of metabolic reprogramming used by T cells, summarize the recent findings of key metabolic pathways and regulators modulating T cell homeostasis, differentiation, and functions in the context of T1D, and discuss the opportunities for metabolic intervention to be employed to suppress autoreactive T cells and limit the progression of β-cell destruction.

## Metabolic Reprogramming of T Cells in T1D

**Figure 1:**
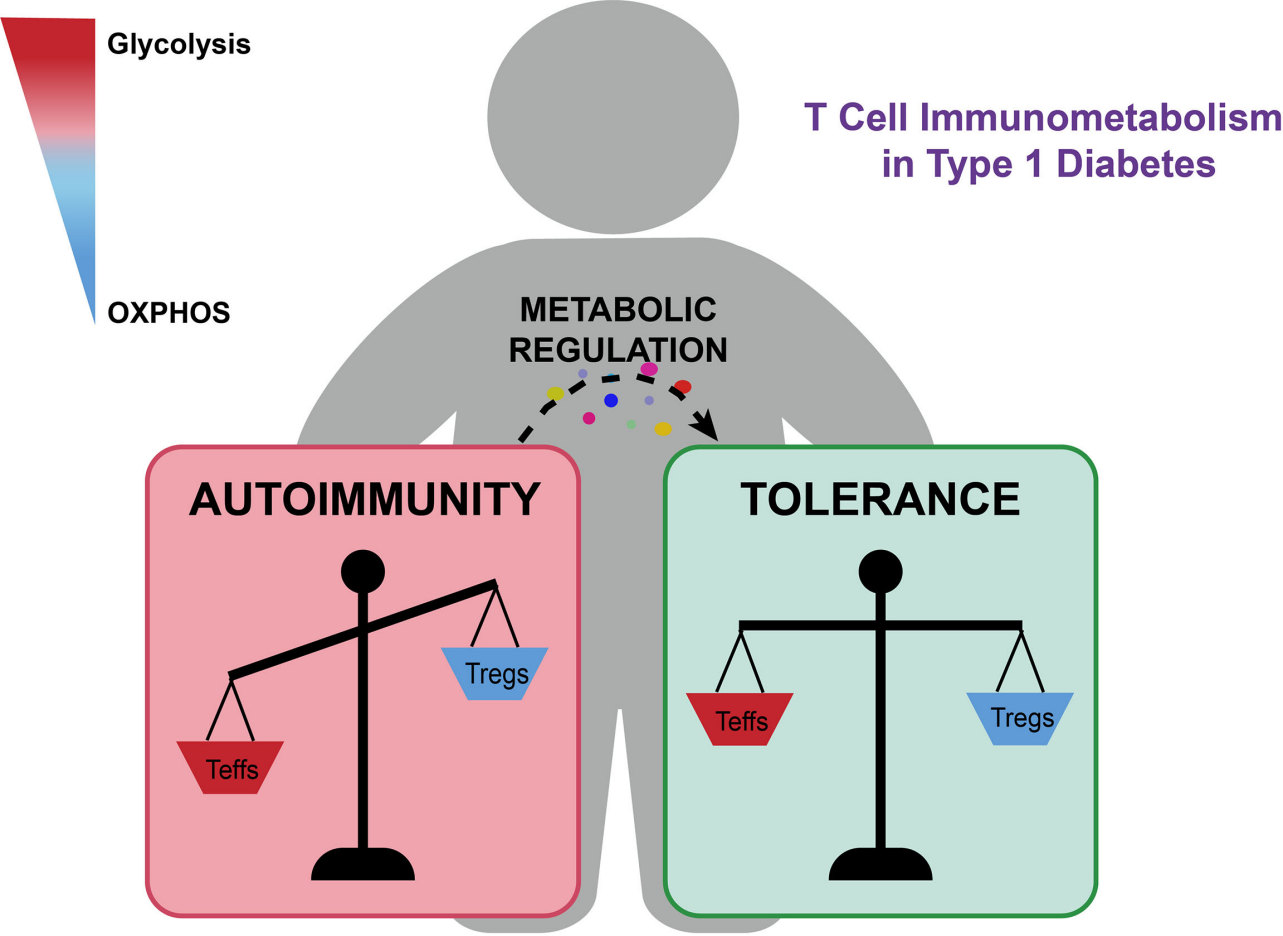
The metabolic imbalance of autoreactive T cells and Tregs contributes to T1D development and progression. Glycolysis supports pro-inflammatory responses, while OXPHOS promotes anti-inflammatory responses and immune tolerance. Immunometabolism regulators of key metabolic pathways, such as metformin and PFK15, could suppress effector T cell responses and facilitate Treg expansion and function, which could provide a promising metabolic intervention strategy for limiting the progression of β-cell destruction and reversing pathologies of T1D.

The pathogenesis of T1D is mainly mediated by the activation of autoreactive CD4+ and CD8+ T cells, which are fueled by metabolic reprogramming (7, 21). Activated effector T cells are more metabolically active and engage mainly in aerobic glycolysis (22). The transition from naïve into effector T cells is driven by variations in anabolic and catabolic metabolism (23). Notably, researchers have reported that glycolysis is essential for cytotoxic T lymphocytes’ function. Treatment of nonobese diabetic (NOD) mice with glycolysis inhibitors resulted in delayed T1D onset and protected β-cell mass (24). In addition, as part of the OXPHOS program, the movement of electrons generates a substantial amount of reactive oxygen species (ROS) (25). The role of ROS in controlling the differentiation of T cells by modulating metabolism has recently been described (21, 25–30). Investigators have shown that ROS can act as signaling molecules involved in the process of T cell activation, proliferation, and function (26). In T1D, ROS generation leads to the activation of autoreactive T cells and β-cell destruction (30). Regulatory T cells (Tregs) are key mediators of peripheral immune tolerance (31–33). Yet, in some autoimmune diseases, Tregs have been shown to have altered stability or function (32). Several researchers have confirmed that impaired Treg function, decreased Treg numbers, or the transition into Th1 (helper T cell 1)-like Treg, contributed to T1D development (33–38). Tregs have unique metabolic preferences that have not been characterized clearly (31, 39). It is generally recognized that Tregs preferentially use OXPHOS and fatty acid oxidation (FAO) for differentiation and function (35, 40, 41)**(** **Figure 1****)**.

## Metabolic Interventions: A New Opportunity for T1D Treatment

Both mammalian target of rapamycin (mTOR) and AMP-activated protein kinase (AMPK) are metabolic sensors required for T cell proliferation and function (19, 42–44). Activation of AMPK inhibits anabolic metabolism, such as nucleic acid and lipid synthesis, but favors catabolic metabolism. In contrast, activation of mTOR signaling facilitates glycolysis, fatty acid production, and mitochondrial biogenesis (42, 45, 46). As mentioned above, autoreactive CD4+/CD8+ T cells exhibit a higher level of glycolysis and depend less on OXPHOS, thus suggesting that glycolysis could be used as an attractive therapeutic target (47). Inhibiting mTOR signaling with rapamycin or enhancing the AMPK signaling pathway with metformin are known to reduce glycolysis (19). Given the key role of AMPK in the activation of T cells, multiple studies have investigated the capacity of metformin to suppress autoimmune diseases, notably T1D. Metformin is now the first line of oral antidiabetic medicine and is used to regulate glucose metabolism (48). Mechanistically, metformin inhibits the mitochondrial electron transport chain (ETC) at Complex I and results in a reduction in intracellular ATP production (48–50). Furthermore, laboratory work demonstrated that metformin could reduce the expansion of activated T cells by inhibiting the expression of cellular myelocytomatosis oncogene (c-Myc) and hypoxia-inducible factor 1 alpha (HIF1-α) in an AMPK-independent way (51–53). Metformin exhibits a dose-dependent effect to control T cell proliferation and suppress the differentiation of Th1 and Th17 cells while enhancing Treg development *in vitro*. NOD mice treated with metformin showed alleviated autoimmune insulitis and reduced amounts of Th1 and Th17 cells in the spleens (50). Furthermore, the anti-inflammatory function of metformin has also been investigated in detail in mouse models of autoimmune arthritis, systemic lupus erythematosus and colitis, all of which portrayed a role of metformin as an anti-inflammatory coordinator and provided the rationale for possible islet protective properties (54–57). Currently, the REMOVAL study and some other smaller trials have proven the clinical advantage of metformin against diverse cardiovascular surrogate endpoints, while the long-term effect of metformin on islet autoimmunity still needs to be further investigated (58–61). As a master regulator of cell metabolism, mTOR has been shown to enhance helper T cell differentiation, especially Th1 and Th17, by modulating glucose metabolism through glucose transporter 1 (Glut1) (62). Therefore, targeting upstream or downstream of mTOR signaling is a potential therapeutic strategy. As a classic mTOR inhibitor, rapamycin decreases the proliferation of Th1 and Th17 cells (63). Furthermore, rapamycin was documented to facilitate Treg expansion and enhance their capability to suppress conventional T cells in a T1D mouse model (34, 64–66). Likewise, augmenting catabolic pathways in CD8+ T cells with metformin or rapamycin decreased the differentiation and proliferation of effector T cells instead of enhancing memory T cell expansion (67). In a phase 2, single-center, randomized, double-blind, placebo-controlled study, rapamycin was shown to decrease insulin requirement in patients with long-term T1D (68). Interestingly, ω-3 polyunsaturated fatty acids (ω-3 PUFAs) have been shown to inhibit CD4+ T cell differentiation *via* suppressing mTOR complex 1 (mTORC1). The pancreatic enrichment of ω-3 PUFAs could inhibit or avoid T1D progression in streptozotocin (STZ)-induced mice (69, 70).

Upon initial activation of lymphocytes, Glut1, one of the typical glucose transporters, is upregulated by the PI3K-Akt-mTOR signaling pathway to enhance glucose influx as well as concomitant with the increased production of key glycolytic enzymes (71, 72). The upregulation of Glut1 is critical for T cell activation, as deletion of Glut1 greatly suppresses proliferation and function of effector T cells (73, 74). Pharmacological blockade of Glut1 might be an efficient way to inhibit autoreactive T cells. The glycolysis inhibitor 2-deoxy-D-glucose (2-DG) is a glucose analog that selectively targets effector T cells with upregulated glycolytic activity (16, 24, 75, 76). NOD mice treated with 2-DG displayed a reduced frequency of activated T cells, decreased immune infiltration within pancreatic islets and increased β-cell granularity (24, 77, 78). Additionally, 2-DG facilitates the differentiation of naïve T cells into Tregs but represses their polarization to Th17 cells (36). Likewise, studies have demonstrated that the combination of 2-DG and metformin reduces CD4+/CD8+ effector T cell responses while inducing Tregs, probably by increasing FAO (79). However, in the light of translation from preclinical trials to clinical application for T1D patients, one of the most relevant side effects of 2-DG is central nervous system toxicity, which demands a prompt solution (80–82). In addition, various natural or synthetic molecules that function as Glut1 inhibitors have emerged in recent years, such as sodium meta-arsenite, STF-31, WZB117 and BAY876, which give us more therapeutic options (73, 83–89).

PFK15, a competitive inhibitor of the rate-limiting glycolysis enzyme, has been found to suppress glycolysis utilization of CD4+ T cells and decrease the response of CD4+ T cells to β-cell antigens. Additionally, treatment of PFK15 in NOD mouse models delayed T1D onset due to metabolic and functional exhaustion of T cells (47). In addition, peroxisome proliferator-activated receptors (PPARs) are transcription factors that control genes involved in glucose and lipid metabolism and FAO (90–92). PPARs are expressed in multiple immune cells including T cells, and modulation of FAO through PPARs provides the possibility to promote immunological intervention therapy (93, 94). Activation of PPARβ/δ inhibits Th1 and Th17 cell differentiation due to the transition from glycolysis to FAO and suppresses the proliferative burst of T cells upon activation (95, 96). Researchers have shown that the PPARα activator fenofibrate and the PPARγ activators troglitazone and rosiglitazone have the capability to decrease the incidence of T1D (95, 97, 98). With the treatment of troglitazone, STZ-induced T1D mice exhibited reduced hyperglycemia and insulitis (99, 100).

Another potential approach to improving T1D is to regulate ROS production. T1D is known to be highly actuated by oxidative stress, as CD4+ T cells require high levels of ROS for optimal activation (26, 101). Utilizing manganese metalloporphyrin (MnP), a ROS scavenger and potent antioxidant, delayed T1D progression through modulating aerobic glycolysis and the mTOR/AMPK axis (102–104). Given the critical role of ROS in autoimmune diseases, researchers have applied superoxide dismutase (SOD) mimetics in a T1D mouse model to promote the longevity and stability of antioxidants to delay β-cell damage (25, 105). T1D was significantly delayed or prevented in NOD mice treated with SOD mimic, partly owing to the decrease in proliferation of CD4+/CD8+ T cells as well as reduced production of pro-inflammatory factors (26, 53, 106, 107). Additionally, lymphocyte activation gene 3 (LAG-3) is an inhibitory receptor expressed on the CD4+ T cell surface, whose deficiency would result in their homeostatic expansion. Studies have reported that the expression of LAG-3 in naïve CD4+ T cells contributes to the restriction of mitochondrial biogenesis and cellular metabolism to keep T cells quiescent. Loss of LAG-3 in NOD mice leads to accelerated T1D progression, potentially by enhancing OXPHOS and glycolytic metabolism and promoting mitochondrial biogenesis of CD4+ T cells (102, 108, 109).

Bacillus Calmette-Guérin (BCG) has been reported as a conducive environmental qualifier of the immune system that could reduce the incidence of autoimmune diseases such as T1D (110). Recent studies indicate that BCG vaccination in patients with long-term T1D showed promising antidiabetic effects, including death of autoimmune T cells as well as expansion of beneficial Tregs (111–113). In an 8-year human study with T1D, BCG vaccination was demonstrated to promote the transition from OXPHOS to aerobic glycolysis of immune cells, improving Treg generation and function, and conferring an immunotolerance effect (114–116). High-mobility group box 1 (HMGB1), an evolutionarily conserved chromosomal protein, was demonstrated to impair the stability and function of Tregs by enhancing PI3K-AKT-mTOR signaling. NOD mice with HMGB1 blockage could protect islet isografts from autoimmune attacks and delay or even reverse T1D development (117).

## Therapeutic Applications of Immunometabolism in Combination Therapy

The complex etiology of T1D is the consequence of failures in controlling autoimmunity as well as perturbations of β cells (118). In addition to controlling autoimmune responses, ideal therapies would also aim to preserve β-cell function and promote β-cell regeneration (119, 120). To date, several immunometabolism-related interventions combined with other therapy regimens have been proven to be successful in NOD mouse models (63, 121–124). For example, the combination treatment regimen of rapamycin and a CD28 antagonist was reported to inhibit T cell activation and migration into pancreatic islets, hence suppressing the progression of T1D (122). Treatment of NOD mice with rapamycin and IL-2 limits T cell expansion and effectively protects islet β-cells from autoimmune attacks (125). Furthermore, combination therapy with rapamycin, islet autoantigen peptides, and IL-2/IL-2 monoclonal antibody complexes increases Treg numbers and protects against autoimmune diabetes in NOD mice (121). However, a phase 1 clinical trial of a rapamycin/IL-2 combination in 10 T1D patients led to transient dysfunction of β cells despite an enrichment of Treg cells (63). Laboratory evidence has demonstrated that IL-21 signaling plays a critical role in promoting lymphocyte infiltration into the pancreas and rewiring T cell metabolism to form long-lived memory CD8+ T cells, which are the predominantly presented T cell subsets in the pancreatic islets of T1D mouse model (126–128). Matthias von Herrath et al. evaluated the combination of immunotherapy (IL-21) and β-cell-directed treatment (liraglutide) in a randomized, double-blind and phase 2 trial in 308 adults with new-onset T1D (129). After fifty-four weeks of treatment and follow-up, C-peptide secretion was prominently improved in the combination therapy group compared with the placebo, but the effect disappeared after therapy cessation in the follow-up period. In conclusion, the effectiveness of combination therapies in animal models and the first large clinical trial provides a promising approach for the development of novel combination therapies (130).

## Conclusion

Our understanding of immunometabolism has considerably advanced over the past few years. Multiple studies have demonstrated that key metabolic enzymes and regulators are involved in different processes of T cell responses by alternating the metabolic pathways and networks to match their specific functional requirements (18, 131, 132). Modulating T cell metabolism has the capability of selectively enhancing or inhibiting particular T cell subsets with distinct functions (133). Of note, although gene knockout mice have presented valuable information, an inescapable limitation is that there are differences between mouse and human immune systems as well as metabolic programs. Moreover, cellular metabolism *in vivo* is distinct from that *in vitro*, while a large number of studies have assessed the metabolism of immune cells during their differentiation, proliferation, and responses *in vitro*. Collectively, targeting T cell metabolism could be a promising strategy for the next wave of immunotherapies treating human diseases, including T1D.

## Author Contributions

JH and BZ: conceptualization and guidance. MZ and YZ wrote and edited the manuscript. All authors contributed to the article and approved the submitted version.

## Funding

This study was supported by the National Natural Science Foundation of China (grant numbers 82170795 and 82100949), and the Outstanding Young Investigator of Hunan Province (2022JJ10094).

## Conflict of Interest

The authors declare that the research was conducted in the absence of any commercial or financial relationships that could be construed as a potential conflict of interest.

## Publisher’s Note

All claims expressed in this article are solely those of the authors and do not necessarily represent those of their affiliated organizations, or those of the publisher, the editors and the reviewers. Any product that may be evaluated in this article, or claim that may be made by its manufacturer, is not guaranteed or endorsed by the publisher.

## References

[1] Norris JM Johnson RK Stene LC. Type 1 Diabetes-Early Life Origins and Changing Epidemiology. Lancet Diabetes Endocrinol (2020) 8:226–38. doi: 10.1016/S2213-8587(19)30412-7 PMC7332108 31999944

[2] Sharp SA Rich SS Wood AR Jones SE Beaumont RN Harrison JW. Development and Standardization of an Improved Type 1 Diabetes Genetic Risk Score for Use in Newborn Screening and Incident Diagnosis. Diabetes Care (2019) 42:200–7. doi: 10.2337/dc18-1785 PMC6341291 30655379

[3] Weng J Zhou Z Guo L Zhu D Ji L Luo X. Incidence of Type 1 Diabetes in China, 2010-13: Population Based Study. BMJ (2018) 360:j5295. doi: 10.1136/bmj.j5295 29298776 PMC5750780

[4] Atkinson MA Eisenbarth GS Michels AW. Type 1 Diabetes. Lancet Lond Engl (2014) 383:69–82. doi: 10.1016/S0140-6736(13)60591-7 PMC4380133 23890997

[5] Chen W Xie A Chan L. Mechanistic Basis of Immunotherapies for Type 1 Diabetes Mellitus. Transl Res J Lab Clin Med (2013) 161:217–29. doi: 10.1016/j.trsl.2012.12.017 PMC3602320 23348026

[6] Stechova K Sklenarova-Labikova J Kratzerova T Pithova P Filipp D. Not Only Glycaemic But Also Other Metabolic Factors Affect T Regulatory Cell Counts and Proinflammatory Cytokine Levels in Women With Type 1 Diabetes. J Diabetes Res (2017) 2017:1–12. doi: 10.1155/2017/5463273 PMC5434466 28553653

[7] Burrack AL Martinov T Fife BT. T Cell-Mediated Beta Cell Destruction: Autoimmunity and Alloimmunity in the Context of Type 1 Diabetes. Front Endocrinol (2017) 8:343. doi: 10.3389/fendo.2017.00343 PMC5723426 29259578

[8] Bluestone JA Buckner JH Fitch M Gitelman SE Gupta S Hellerstein MK. Type 1 Diabetes Immunotherapy Using Polyclonal Regulatory T Cells. Sci Transl Med (2015) 7(315):315ra189. doi: 10.1126/scitranslmed.aad4134 PMC4729454 26606968

[9] Jacobsen LM Newby BN Perry DJ Posgai AL Haller MJ Brusko TM. Immune Mechanisms and Pathways Targeted in Type 1 Diabetes. Curr Diabetes Rep (2018) 18:90. doi: 10.1007/s11892-018-1066-5 PMC8053389 30168021

[10] Smith EL Peakman M. Peptide Immunotherapy for Type 1 Diabetes-Clinical Advances. Front Immunol (2018) 9:392. doi: 10.3389/fimmu.2018.00392 29541078 PMC5836708

[11] Bone RN Evans-Molina C. Combination Immunotherapy for Type 1 Diabetes. Curr Diabetes Rep (2017) 17:50. doi: 10.1007/s11892-017-0878-z PMC5774222 28534310

[12] Huang H Zhou P Wei J Long L Shi H Dhungana Y. *In Vivo* CRISPR Screening Reveals Nutrient Signaling Processes Underpinning CD8+ T Cell Fate Decisions. Cell (2021) 184:1245–1261.e21. doi: 10.1016/j.cell.2021.02.021 33636132 PMC8101447

[13] Ceballos B Alexander M Lakey JRT. Advanced Approaches in Immunotherapy for the Treatment of Type 1 Diabetes Mellitus. EMJ Diabetes (2020). doi: 10.33590/emjdiabet/20-00062

[14] Lee M-S Bensinger SJ. Reprogramming Cholesterol Metabolism in Macrophages and Its Role in Host Defense Against Cholesterol-Dependent Cytolysins. Cell Mol Immunol (2022) 19:327–36. doi: 10.1038/s41423-021-00827-0 PMC8891295 35017717

[15] Chi H. Immunometabolism at the Intersection of Metabolic Signaling, Cell Fate, and Systems Immunology. Cell Mol Immunol (2022) 19:299–302. doi: 10.1038/s41423-022-00840-x 35190684 PMC8891332

[16] Lercher A Baazim H Bergthaler A. Systemic Immunometabolism: Challenges and Opportunities. Immunity (2020) 53:496–509. doi: 10.1016/j.immuni.2020.08.012 32937151 PMC7491485

[17] Martins CP Piganelli J. Targeting T Cell Metabolism to Combat Autoimmunity: Implications for the Future of Type 1 Diabetes Therapeutics. (2020). doi: 10.20900/immunometab20200010

[18] Nm C HC. Metabolic Adaptation of Lymphocytes in Immunity and Disease. Immunity (2022) 55(1):14–30. doi: 10.1016/j.immuni.2021.12.012 35021054 PMC8842882

[19] Galgani M De Rosa V Matarese G. T Cell Metabolism and Susceptibility to Autoimmune Diseases. Mol Immunol (2015) 68:558–63. doi: 10.1016/j.molimm.2015.07.035 26265113

[20] Pålsson-McDermott EM O’Neill LAJ. Targeting Immunometabolism as an Anti-Inflammatory Strategy. Cell Res (2020) 30:300–14. doi: 10.1038/s41422-020-0291-z PMC7118080 32132672

[21] Previte DM Piganelli JD. Reactive Oxygen Species and Their Implications on CD4+ T Cells in Type 1 Diabetes. Antioxid Redox Signal (2018) 29:1399–414. doi: 10.1089/ars.2017.7357 28990401

[22] Dimeloe S Burgener A Grählert J Hess C. T-Cell Metabolism Governing Activation, Proliferation and Differentiation; a Modular View. Immunology (2017) 150:35–44. doi: 10.1111/imm.12655 27479920 PMC5341500

[23] Chen H Yang T Zhu L Zhao Y. Cellular Metabolism on T-Cell Development and Function. Int Rev Immunol (2015) 34:19–33. doi: 10.3109/08830185.2014.902452 24708060

[24] Garyu JW Uduman M Stewart A Rui J Deng S Shenson J. Characterization Of Diabetogenic Cd8+ T Cells: Immune Therapy With Metabolic Blockade. J Biol Chem (2016) 291:11230–40. doi: 10.1074/jbc.M115.713362 PMC4900270 26994137

[25] Neyestani TR Ghandchi Z Eshraghian M-R Kalayi A Shariatzadeh N Houshiarrad A. Evidence for Augmented Oxidative Stress in the Subjects With Type 1 Diabetes and Their Siblings: A Possible Preventive Role for Antioxidants. Eur J Clin Nutr (2012) 66:1054–8. doi: 10.1038/ejcn.2012.81 22781023

[26] Chávez MD Tse HM. Targeting Mitochondrial-Derived Reactive Oxygen Species in T Cell-Mediated Autoimmune Diseases. Front Immunol (2021) 12:703972. doi: 10.3389/fimmu.2021.703972 34276700 PMC8281042

[27] Buck MD O’Sullivan D Klein Geltink RI Curtis JD Chang C-H Sanin DE. Mitochondrial Dynamics Controls T Cell Fate Through Metabolic Programming. Cell (2016) 166:63–76. doi: 10.1016/j.cell.2016.05.035 27293185 PMC4974356

[28] Klein Geltink RI O’Sullivan D Corrado M Bremser A Buck MD Buescher JM. Mitochondrial Priming by CD28. Cell (2017) 171:385–397.e11. doi: 10.1016/j.cell.2017.08.018 28919076 PMC5637396

[29] Chen J Stimpson SE Fernandez-Bueno GA Mathews CE. Mitochondrial Reactive Oxygen Species and Type 1 Diabetes. Antioxid Redox Signal (2018) 29:1361–72. doi: 10.1089/ars.2017.7346 PMC6166689 29295631

[30] Padgett LE Broniowska KA Hansen PA Corbett JA Tse HM. The Role of Reactive Oxygen Species and Proinflammatory Cytokines in Type 1 Diabetes Pathogenesis. Ann N Y Acad Sci (2013) 1281:16–35. doi: 10.1111/j.1749-6632.2012.06826.x 23323860 PMC3715103

[31] Kitz A Singer E Hafler D. Regulatory T Cells: From Discovery to Autoimmunity. Cold Spring Harb Perspect Med (2018) 8:a029041. doi: 10.1101/cshperspect.a029041 29311129 PMC6280708

[32] Hua J Inomata T Chen Y Foulsham W Stevenson W Shiang T. Pathological Conversion of Regulatory T Cells is Associated With Loss of Allotolerance. Sci Rep (2018) 8:7059. doi: 10.1038/s41598-018-25384-x 29728574 PMC5935752

[33] Visperas A Vignali DAA. Are Regulatory T Cells Defective in Type 1 Diabetes and Can We Fix Them? J Immunol Baltim Md 1950 (2016) 197:3762–70. doi: 10.4049/jimmunol.1601118 PMC5119643 27815439

[34] Galgani M De Rosa V La Cava A Matarese G. Role of Metabolism in the Immunobiology of Regulatory T Cells. J Immunol Baltim Md 1950 (2016) 197:2567–75. doi: 10.4049/jimmunol.1600242 PMC5027986 27638939

[35] Newton R Priyadharshini B Turka LA. Immunometabolism of Regulatory T Cells. Nat Immunol (2016) 17:618–25. doi: 10.1038/ni.3466 PMC5006394 27196520

[36] Tanimine N Germana SK Fan M Hippen K Blazar BR Markmann JF. Differential Effects of 2-Deoxy-D-Glucose on *In Vitro* Expanded Human Regulatory T Cell Subsets. PloS One (2019) 14:e0217761. doi: 10.1371/journal.pone.0217761 31170216 PMC6553739

[37] Chen Y Chen S Gu Y Feng Y Shi Y Fu Q. CTLA-4 +49 G/A, a Functional T1D Risk SNP, Affects CTLA-4 Level in Treg Subsets and IA-2A Positivity, But Not Beta-Cell Function. Sci Rep (2018) 8:10074. doi: 10.1038/s41598-018-28423-9 29973665 PMC6031668

[38] Lawson JM Tremble J Dayan C Beyan H Leslie RDG Peakman M. Increased Resistance to CD4+CD25hi Regulatory T Cell-Mediated Suppression in Patients With Type 1 Diabetes. Clin Exp Immunol (2008) 154:353–9. doi: 10.1111/j.1365-2249.2008.03810.x PMC2633239 19037920

[39] Tsutsumi Y Jie X Ihara K Nomura A Kanemitsu S Takada H. Phenotypic and Genetic Analyses of T-Cell-Mediated Immunoregulation in Patients With Type 1 Diabetes. Diabetes Med J Br Diabetes Assoc (2006) 23:1145–50. doi: 10.1111/j.1464-5491.2006.01951.x 16978382

[40] Michalek RD Gerriets VA Jacobs SR Macintyre AN MacIver NJ Mason EF. Cutting Edge: Distinct Glycolytic and Lipid Oxidative Metabolic Programs are Essential for Effector and Regulatory CD4+ T Cell Subsets. J Immunol Baltim Md 1950 (2011) 186:3299–303. doi: 10.4049/jimmunol.1003613 PMC3198034 21317389

[41] Angelin A Gil-de-Gómez L Dahiya S Jiao J Guo L Levine MH. Foxp3 Reprograms T Cell Metabolism to Function in Low-Glucose, High-Lactate Environments. Cell Metab (2017) 25:1282–1293.e7. doi: 10.1016/j.cmet.2016.12.018 28416194 PMC5462872

[42] Zeng H Yang K Cloer C Neale G Vogel P Chi H. Mtorc1 Couples Immune Signals and Metabolic Programming to Establish T(reg)-Cell Function. Nature (2013) 499:485–90. doi: 10.1038/nature12297 PMC3759242 23812589

[43] Sun I-H Oh M-H Zhao L Patel CH Arwood ML Xu W. mTOR Complex 1 Signaling Regulates the Generation and Function of Central and Effector Foxp3+ Regulatory T Cells. J Immunol Baltim Md 1950 (2018) 201:481–92. doi: 10.4049/jimmunol.1701477 PMC6089237 29884702

[44] Chou W-C Rampanelli E Li X Ting JP-Y. Impact of Intracellular Innate Immune Receptors on Immunometabolism. Cell Mol Immunol (2022) 19:337–51. doi: 10.1038/s41423-021-00780-y PMC8891342 34697412

[45] Pollizzi KN Powell JD. Regulation of T Cells by mTOR: The Known Knowns and the Known Unknowns. Trends Immunol (2015) 36:13–20. doi: 10.1016/j.it.2014.11.005 25522665 PMC4290883

[46] Yang K Shrestha S Zeng H Karmaus PWF Neale G Vogel P. T Cell Exit From Quiescence and Differentiation Into Th2 Cells Depend on Raptor-Mtorc1-Mediated Metabolic Reprogramming. Immunity (2013) 39:1043–56. doi: 10.1016/j.immuni.2013.09.015 PMC3986063 24315998

[47] Martins CP New LA O’Connor EC Previte DM Cargill KR Tse IL. Glycolysis Inhibition Induces Functional and Metabolic Exhaustion of CD4+ T Cells in Type 1 Diabetes. Front Immunol (2021) 12:669456. doi: 10.3389/fimmu.2021.669456 34163475 PMC8216385

[48] Zhou G Myers R Li Y Chen Y Shen X Fenyk-Melody J. Role of AMP-Activated Protein Kinase in Mechanism of Metformin Action. J Clin Invest (2001) 108:1167–74. doi: 10.1172/JCI13505 PMC209533 11602624

[49] Stephenne X Foretz M Taleux N van der Zon GC Sokal E Hue L. Metformin Activates AMP-Activated Protein Kinase in Primary Human Hepatocytes by Decreasing Cellular Energy Status. Diabetologia (2011) 54:3101–10. doi: 10.1007/s00125-011-2311-5 PMC3210354 21947382

[50] Duan W Ding Y Yu X Ma D Yang B Li Y. Metformin Mitigates Autoimmune Insulitis by Inhibiting Th1 and Th17 Responses While Promoting Treg Production. Am J Transl Res (2019) 11(4):2393–402. PMC6511786 31105845

[51] Foretz M Hébrard S Leclerc J Zarrinpashneh E Soty M Mithieux G. Metformin Inhibits Hepatic Gluconeogenesis in Mice Independently of the LKB1/AMPK Pathway *via* a Decrease in Hepatic Energy State. J Clin Invest (2010) 120:2355–69. doi: 10.1172/JCI40671 PMC2898585 20577053

[52] Kim YD Park K-G Lee Y-S Park Y-Y Kim D-K Nedumaran B. Metformin Inhibits Hepatic Gluconeogenesis Through AMP-Activated Protein Kinase-Dependent Regulation of the Orphan Nuclear Receptor SHP. Diabetes (2008) 57:306–14. doi: 10.2337/db07-0381 17909097

[53] Zarrouk M Finlay DK Foretz M Viollet B Cantrell DA. Adenosine-Mono-Phosphate-Activated Protein Kinase-Independent Effects of Metformin in T Cells. PloS One (2014) 9:e106710. doi: 10.1371/journal.pone.0106710 25181053 PMC4152329

[54] de Bari L Atlante A. Including the Mitochondrial Metabolism of L-Lactate in Cancer Metabolic Reprogramming. Cell Mol Life Sci CMLS (2018) 75:2763–76. doi: 10.1007/s00018-018-2831-y PMC11105303 29728715

[55] Lee S-Y Lee SH Yang E-J Kim E-K Kim J-K Shin D-Y. Metformin Ameliorates Inflammatory Bowel Disease by Suppression of the STAT3 Signaling Pathway and Regulation of the Between Th17/Treg Balance. PloS One (2015) 10:e0135858. doi: 10.1371/journal.pone.0135858 26360050 PMC4567351

[56] Kim EK Lee SH Jhun JY Byun JK Jeong JH Lee S-Y. Metformin Prevents Fatty Liver and Improves Balance of White/Brown Adipose in an Obesity Mouse Model by Inducing Fgf21. Mediators Inflammation (2016) 2016:5813030. doi: 10.1155/2016/5813030 PMC4745345 27057099

[57] Yin Y Choi S-C Xu Z Perry DJ Seay H Croker BP. Normalization of CD4+ T Cell Metabolism Reverses Lupus. Sci Transl Med (2015) 7:274ra18. doi: 10.1126/scitranslmed.aaa0835 PMC5292723 25673763

[58] Snaith JR Holmes-Walker DJ Greenfield JR. Reducing Type 1 Diabetes Mortality: Role for Adjunctive Therapies? Trends Endocrinol Metab (2020) 31:150–64. doi: 10.1016/j.tem.2019.11.007 31822381

[59] Vella S Buetow L Royle P Livingstone S Colhoun HM Petrie JR. The Use of Metformin in Type 1 Diabetes: A Systematic Review of Efficacy. Diabetologia (2010) 53:809–20. doi: 10.1007/s00125-009-1636-9 20057994

[60] Standl E. Metformin in Type 1 Diabetes. Lancet Diabetes Endocrinol (2017) 5:567–9. doi: 10.1016/S2213-8587(17)30216-4 28615150

[61] Sciannimanico S Grimaldi F Vescini F De Pergola G Iacoviello M Licchelli B. Metformin: Up to Date. Endocr Metab Immune Disord - Drug Targets (2020) 20:172–81. doi: 10.2174/1871530319666190507125847 31670618

[62] Waickman AT Powell JD. mTOR, Metabolism, and the Regulation of T-Cell Differentiation and Function. Immunol Rev (2012) 249:43–58. doi: 10.1111/j.1600-065X.2012.01152.x 22889214 PMC3419491

[63] Long SA Rieck M Sanda S Bollyky JB Samuels PL Goland R. Rapamycin/IL-2 Combination Therapy in Patients With Type 1 Diabetes Augments Tregs Yet Transiently Impairs β-Cell Function. Diabetes (2012) 61:2340–8. doi: 10.2337/db12-0049 PMC3425404 22721971

[64] Battaglia M Stabilini A Migliavacca B Horejs-Hoeck J Kaupper T Roncarolo M-G. Rapamycin Promotes Expansion of Functional CD4+CD25+FOXP3+ Regulatory T Cells of Both Healthy Subjects and Type 1 Diabetic Patients. J Immunol Baltim Md 1950 (2006) 177:8338–47. doi: 10.4049/jimmunol.177.12.8338 17142730

[65] Monti P Scirpoli M Maffi P Piemonti L Secchi A Bonifacio E. Rapamycin Monotherapy in Patients With Type 1 Diabetes Modifies CD4+CD25+FOXP3+ Regulatory T-Cells. Diabetes (2008) 57:2341–7. doi: 10.2337/db08-0138 PMC2518485 18559659

[66] Battaglia M Stabilini A Draghici E Migliavacca B Gregori S Bonifacio E. Induction of Tolerance in Type 1 Diabetes *via* Both CD4+CD25+ T Regulatory Cells and T Regulatory Type 1 Cells. Diabetes (2006) 55:1571–80. doi: 10.2337/db05-1576 16731819

[67] Pearce EL Walsh MC Cejas PJ Harms GM Shen H Wang L-S. Enhancing CD8 T-Cell Memory by Modulating Fatty Acid Metabolism. Nature (2009) 460:103–7. doi: 10.1038/nature08097 PMC2803086 19494812

[68] Bolla AM Gandolfi A Borgonovo E Laurenzi A Caretto A Molinari C. Rapamycin Plus Vildagliptin to Recover β-Cell Function in Long-Standing Type 1 Diabetes: A Double-Blind, Randomized Trial. J Clin Endocrinol Metab (2021) 106:e507–19. doi: 10.1210/clinem/dgaa791 33124663

[69] Li X Bi X Wang S Zhang Z Li F Zhao AZ. Therapeutic Potential of ω-3 Polyunsaturated Fatty Acids in Human Autoimmune Diseases. Front Immunol (2019) 10:2241. doi: 10.3389/fimmu.2019.02241 31611873 PMC6776881

[70] Bellenger J Bellenger S Bataille A Massey KA Nicolaou A Rialland M. High Pancreatic N-3 Fatty Acids Prevent STZ-Induced Diabetes in Fat-1 Mice: Inflammatory Pathway Inhibition. Diabetes (2011) 60:1090–9. doi: 10.2337/db10-0901 PMC3064083 21330635

[71] Swainson L Kinet S Manel N Battini J-L Sitbon M Taylor N. Glucose Transporter 1 Expression Identifies a Population of Cycling CD4 ^+^ CD8 ^+^ Human Thymocytes With High CXCR4-Induced Chemotaxis. Proc Natl Acad Sci (2005) 102:12867–72. doi: 10.1073/pnas.0503603102 PMC1200272 16126902

[72] Wang R Dillon CP Shi LZ Milasta S Carter R Finkelstein D. The Transcription Factor Myc Controls Metabolic Reprogramming Upon T Lymphocyte Activation. Immunity (2011) 35:871–82. doi: 10.1016/j.immuni.2011.09.021 PMC3248798 22195744

[73] Di Dedda C Vignali D Piemonti L Monti P. Pharmacological Targeting of GLUT1 to Control Autoreactive T Cell Responses. Int J Mol Sci (2019) 20:4962. doi: 10.3390/ijms20194962 PMC6801424 31597342

[74] Macintyre AN Gerriets VA Nichols AG Michalek RD Rudolph MC Deoliveira D. The Glucose Transporter Glut1 is Selectively Essential for CD4 T Cell Activation and Effector Function. Cell Metab (2014) 20:61–72. doi: 10.1016/j.cmet.2014.05.004 24930970 PMC4079750

[75] Apaya MK Kuo T-F Yang M-T Yang G Hsiao C-L Chang S-B. Phytochemicals as Modulators of β-Cells and Immunity for the Therapy of Type 1 Diabetes: Recent Discoveries in Pharmacological Mechanisms and Clinical Potential. Pharmacol Res (2020) 156:104754. doi: 10.1016/j.phrs.2020.104754 32173584

[76] Zhang D Li J Wang F Hu J Wang S Sun Y. 2-Deoxy-D-Glucose Targeting of Glucose Metabolism in Cancer Cells as a Potential Therapy. Cancer Lett (2014) 355:176–83. doi: 10.1016/j.canlet.2014.09.003 25218591

[77] Sukumar M Liu J Ji Y Subramanian M Crompton JG Yu Z. Inhibiting Glycolytic Metabolism Enhances CD8+ T Cell Memory and Antitumor Function. J Clin Invest (2013) 123:4479–88. doi: 10.1172/JCI69589 PMC3784544 24091329

[78] Cham CM Driessens G O’Keefe JP Gajewski TF. Glucose Deprivation Inhibits Multiple Key Gene Expression Events and Effector Functions in CD8+ T Cells. Eur J Immunol (2008) 38:2438–50. doi: 10.1002/eji.200838289 PMC3008428 18792400

[79] Bordignon C Canu A Dyczko A Leone S Monti P. T-Cell Metabolism as a Target to Control Autoreactive T Cells in β-Cell Autoimmunity. Curr Diabetes Rep (2017) 17:24. doi: 10.1007/s11892-017-0848-5 PMC5355511 28303386

[80] Leen WG Taher M Verbeek MM Kamsteeg EJ van de Warrenburg BP Willemsen MA. GLUT1 Deficiency Syndrome Into Adulthood: A Follow-Up Study. J Neurol (2014) 261:589–99. doi: 10.1007/s00415-014-7240-z 24413642

[81] De Giorgis V Veggiotti P. GLUT1 Deficiency Syndrome 2013: Current State of the Art. Seizure (2013) 22:803–11. doi: 10.1016/j.seizure.2013.07.003 23890838

[82] Pearson TS Akman C Hinton VJ Engelstad K De Vivo DC. Phenotypic Spectrum of Glucose Transporter Type 1 Deficiency Syndrome (Glut1 Ds). Curr Neurol Neurosci Rep (2013) 13:342. doi: 10.1007/s11910-013-0342-7 23443458

[83] Liu Y Cao Y Zhang W Bergmeier S Qian Y Akbar H. A Small-Molecule Inhibitor of Glucose Transporter 1 Downregulates Glycolysis, Induces Cell-Cycle Arrest, and Inhibits Cancer Cell Growth *In Vitro* and *In Vivo* . Mol Cancer Ther (2012) 11:1672–82. doi: 10.1158/1535-7163.MCT-12-0131 22689530

[84] Vignali D Cantarelli E Bordignon C Canu A Citro A Annoni A. Detection and Characterization of CD8+ Autoreactive Memory Stem T Cells in Patients With Type 1 Diabetes. Diabetes (2018) 67:936–45. doi: 10.2337/db17-1390 29506985

[85] Siebeneicher H Cleve A Rehwinkel H Neuhaus R Heisler I Müller T. Identification and Optimization of the First Highly Selective GLUT1 Inhibitor BAY-876. ChemMedChem (2016) 11:2261–71. doi: 10.1002/cmdc.201600276 PMC5095872 27552707

[86] Adams DJ Ito D Rees MG Seashore-Ludlow B Puyang X Ramos AH. NAMPT Is the Cellular Target of STF-31-Like Small-Molecule Probes. ACS Chem Biol (2014) 9:2247–54. doi: 10.1021/cb500347p PMC4201331 25058389

[87] Ma Y Wang W Idowu MO Oh U Wang X-Y Temkin SM. Ovarian Cancer Relies on Glucose Transporter 1 to Fuel Glycolysis and Growth: Anti-Tumor Activity of BAY-876. Cancers (2018) 11:E33. doi: 10.3390/cancers11010033 30602670 PMC6356953

[88] Ojelabi OA Lloyd KP Simon AH De Zutter JK Carruthers A. WZB117 (2-Fluoro-6-(M-Hydroxybenzoyloxy) Phenyl M-Hydroxybenzoate) Inhibits GLUT1-Mediated Sugar Transport by Binding Reversibly at the Exofacial Sugar Binding Site. J Biol Chem (2016) 291:26762–72. doi: 10.1074/jbc.M116.759175 PMC5207184 27836974

[89] Lee YS Kim D Lee EK Kim S Choi CS Jun HS. Sodium Meta-Arsenite Prevents the Development of Autoimmune Diabetes in NOD Mice. Toxicol Appl Pharmacol (2015) 284:254–61. doi: 10.1016/j.taap.2014.12.016 25576766

[90] Wahli W Michalik L. PPARs at the Crossroads of Lipid Signaling and Inflammation. Trends Endocrinol Metab TEM (2012) 23:351–63. doi: 10.1016/j.tem.2012.05.001 22704720

[91] Gross B Pawlak M Lefebvre P Staels B. PPARs in Obesity-Induced T2DM, Dyslipidaemia and NAFLD. Nat Rev Endocrinol (2017) 13:36–49. doi: 10.1038/nrendo.2016.135 27636730

[92] Zhang S Yang X Luo J Ge X Sun W Zhu H. Pparα Activation Sensitizes Cancer Cells to Epigallocatechin-3-Gallate (EGCG) Treatment *via* Suppressing Heme Oxygenase-1. Nutr Cancer (2014) 66:315–24. doi: 10.1080/01635581.2014.868909 24447094

[93] Holm LJ Mønsted MØ Haupt-Jorgensen M Buschard K. PPARs and the Development of Type 1 Diabetes. PPAR Res (2020) 2020:1–11. doi: 10.1155/2020/6198628 PMC7199578 32395123

[94] Cariello M Piccinin E Moschetta A. Transcriptional Regulation of Metabolic Pathways *via* Lipid-Sensing Nuclear Receptors PPARs, FXR, and LXR in NASH. Cell Mol Gastroenterol Hepatol (2021) 11:1519–39. doi: 10.1016/j.jcmgh.2021.01.012 PMC8042405 33545430

[95] Fu Z Zhen W Yuskavage J Liu D. Epigallocatechin Gallate Delays the Onset of Type 1 Diabetes in Spontaneous non-Obese Diabetic Mice. Br J Nutr (2011) 105:1218–25. doi: 10.1017/S0007114510004824 PMC4335671 21144096

[96] Holm LJ Krogvold L Hasselby JP Kaur S Claessens LA Russell MA. Abnormal Islet Sphingolipid Metabolism in Type 1 Diabetes. Diabetologia (2018) 61:1650–61. doi: 10.1007/s00125-018-4614-2 PMC6445476 29671030

[97] Beales PE Pozzilli P. Thiazolidinediones for the Prevention of Diabetes in the non-Obese Diabetic (NOD) Mouse: Implications for Human Type 1 Diabetes. Diabetes Metab Res Rev (2002) 18:114–7. doi: 10.1002/dmrr.262 11994902

[98] Ogawa J Takahashi S Fujiwara T Fukushige J Hosokawa T Izumi T. Troglitazone can Prevent Development of Type 1 Diabetes Induced by Multiple Low-Dose Streptozotocin in Mice. Life Sci (1999) 65:1287–96. doi: 10.1016/s0024-3205(99)00364-1 10503944

[99] Weiss L Zeira M Reich S Har-Noy M Mechoulam R Slavin S. Cannabidiol Lowers Incidence of Diabetes in non-Obese Diabetic Mice. Autoimmunity (2006) 39:143–51. doi: 10.1080/08916930500356674 16698671

[100] Castro CN Barcala Tabarrozzi AE Winnewisser J Gimeno ML Antunica Noguerol M Liberman AC. Curcumin Ameliorates Autoimmune Diabetes. Evidence in Accelerated Murine Models of Type 1 Diabetes. Clin Exp Immunol (2014) 177:149–60. doi: 10.1111/cei.12322 PMC4089164 24628444

[101] Chen J Chernatynskaya AV Li J-W Kimbrell MR Cassidy RJ Perry DJ. T Cells Display Mitochondria Hyperpolarization in Human Type 1 Diabetes. Sci Rep (2017) 7:10835. doi: 10.1038/s41598-017-11056-9 28883439 PMC5589742

[102] Previte DM O’Connor EC Novak EA Martins CP Mollen KP Piganelli JD. Reactive Oxygen Species are Required for Driving Efficient and Sustained Aerobic Glycolysis During CD4+ T Cell Activation. PloS One (2017) 12:e0175549. doi: 10.1371/journal.pone.0175549 28426686 PMC5398529

[103] Delmastro-Greenwood MM Tse HM Piganelli JD. Effects of Metalloporphyrins on Reducing Inflammation and Autoimmunity. Antioxid Redox Signal (2014) 20:2465–77. doi: 10.1089/ars.2013.5257 23472672

[104] Delmastro-Greenwood MM Votyakova T Goetzman E Marre ML Previte DM Tovmasyan A. Mn Porphyrin Regulation of Aerobic Glycolysis: Implications on the Activation of Diabetogenic Immune Cells. Antioxid Redox Signal (2013) 19:1902–15. doi: 10.1089/ars.2012.5167 PMC3931434 23682840

[105] Coudriet GM Delmastro-Greenwood MM Previte DM Marré ML O’Connor EC Novak EA. Treatment With a Catalytic Superoxide Dismutase (SOD) Mimetic Improves Liver Steatosis, Insulin Sensitivity, and Inflammation in Obesity-Induced Type 2 Diabetes. Antioxid Basel Switz (2017) 6:E85. doi: 10.3390/antiox6040085 PMC5745495 29104232

[106] Sklavos MM Tse HM Piganelli JD. Redox Modulation Inhibits CD8 T Cell Effector Function. Free Radic Biol Med (2008) 45:1477–86. doi: 10.1016/j.freeradbiomed.2008.08.023 18805480

[107] Piganelli JD Flores SC Cruz C Koepp J Batinic-Haberle I Crapo J. A Metalloporphyrin-Based Superoxide Dismutase Mimic Inhibits Adoptive Transfer of Autoimmune Diabetes by a Diabetogenic T-Cell Clone. Diabetes (2002) 51:347–55. doi: 10.2337/diabetes.51.2.347 11812741

[108] Previte DM Martins CP O’Connor EC Marre ML Coudriet GM Beck NW. Lymphocyte Activation Gene-3 Maintains Mitochondrial and Metabolic Quiescence in Naive CD4+ T Cells. Cell Rep (2019) 27:129–141.e4. doi: 10.1016/j.celrep.2019.03.004 30943396

[109] Mouat IC Morse ZJ Jean-Baptiste VSE Allanach JR Horwitz MS. Fresh Ideas, Foundational Experiments (FIFE): Immunology and Diabetes 2016 FIFE Symposium. Front Endocrinol (2017) 8:238. doi: 10.3389/fendo.2017.00238 PMC5610696 28974943

[110] Singh AK Netea MG Bishai WR. BCG Turns 100: Its Nontraditional Uses Against Viruses, Cancer, and Immunologic Diseases. J Clin Invest (2021) 131:e148291. doi: 10.1172/JCI148291 PMC8159679 34060492

[111] Huppmann M Baumgarten A Ziegler A-G Bonifacio E. Neonatal Bacille Calmette-Guerin Vaccination and Type 1 Diabetes. Diabetes Care (2005) 28:1204–6. doi: 10.2337/diacare.28.5.1204 15855590

[112] Faustman DL. Benefits of BCG-Induced Metabolic Switch From Oxidative Phosphorylation to Aerobic Glycolysis in Autoimmune and Nervous System Diseases. J Intern Med (2020) 288:641–50. doi: 10.1111/joim.13050 32107806

[113] Faustman DL Wang L Okubo Y Burger D Ban L Man G. Proof-Of-Concept, Randomized, Controlled Clinical Trial of Bacillus-Calmette-Guerin for Treatment of Long-Term Type 1 Diabetes. PloS One (2012) 7:e41756. doi: 10.1371/journal.pone.0041756 22905105 PMC3414482

[114] De Rosa V Galgani M Porcellini A Colamatteo A Santopaolo M Zuchegna C. Glycolysis Controls the Induction of Human Regulatory T Cells by Modulating the Expression of FOXP3 Exon 2 Splicing Variants. Nat Immunol (2015) 16:1174–84. doi: 10.1038/ni.3269 PMC4868085 26414764

[115] Kühtreiber WM Tran L Kim T Dybala M Nguyen B Plager S. Long-Term Reduction in Hyperglycemia in Advanced Type 1 Diabetes: The Value of Induced Aerobic Glycolysis With BCG Vaccinations. NPJ Vaccines (2018) 3:23. doi: 10.1038/s41541-018-0062-8 29951281 PMC6013479

[116] Faustman DL. TNF, TNF Inducers, and TNFR2 Agonists: A New Path to Type 1 Diabetes Treatment. Diabetes Metab Res Rev (2018) 34(1). doi: 10.1002/dmrr.2941 28843039

[117] Zhang J Chen L Wang F Zou Y Li J Luo J. Extracellular HMGB1 Exacerbates Autoimmune Progression and Recurrence of Type 1 Diabetes by Impairing Regulatory T Cell Stability. Diabetologia (2020) 63:987–1001. doi: 10.1007/s00125-020-05105-8 32072192 PMC7145789

[118] Campbell-Thompson M Fu A Kaddis JS Wasserfall C Schatz DA Pugliese A. Insulitis and β-Cell Mass in the Natural History of Type 1 Diabetes. Diabetes (2016) 65:719–31. doi: 10.2337/db15-0779 PMC4764143 26581594

[119] Matthews JB Staeva TP Bernstein PL Peakman M Von Herrath M. Developing Combination Immunotherapies for Type 1 Diabetes: Recommendations From the ITN–JDRF Type 1 Diabetes Combination Therapy Assessment Group. Clin Exp Immunol (2010) 160:176–84. doi: 10.1111/j.1365-2249.2010.04153.x PMC2857940 20629979

[120] von Scholten BJ Kreiner FF Gough SCL von Herrath M. Current and Future Therapies for Type 1 Diabetes. Diabetologia (2021) 64:1037–48. doi: 10.1007/s00125-021-05398-3 PMC8012324 33595677

[121] Manirarora JN Wei C-H. Combination Therapy Using IL-2/IL-2 Monoclonal Antibody Complexes, Rapamycin, and Islet Autoantigen Peptides Increases Regulatory T Cell Frequency and Protects Against Spontaneous and Induced Type 1 Diabetes in Nonobese Diabetic Mice. J Immunol Baltim Md 1950 (2015) 195:5203–14. doi: 10.4049/jimmunol.1402540 26482409

[122] Besançon A Goncalves T Valette F Mary C Vanhove B Chatenoud L. A Selective CD28 Antagonist and Rapamycin Synergise to Protect Against Spontaneous Autoimmune Diabetes in NOD Mice. Diabetologia (2018) 61:1811–6. doi: 10.1007/s00125-018-4638-7 29845333

[123] Chen N Kroger CJ Tisch RM Bachelder EM Ainslie KM. Prevention of Type 1 Diabetes With Acetalated Dextran Microparticles Containing Rapamycin and Pancreatic Peptide P31. Adv Healthc Mater (2018) 7:e1800341. doi: 10.1002/adhm.201800341 30051618

[124] Guclu M Oz Gul O Cander S Unal O Ozkaya G Sarandol E. Effect of Rosiglitazone and Insulin Combination Therapy on Inflammation Parameters and Adipocytokine Levels in Patients With Type 1 Dm. J Diabetes Res (2015) 2015:807891. doi: 10.1155/2015/807891 26273677 PMC4530282

[125] Rabinovitch A Suarez-Pinzon WL Shapiro AMJ Rajotte RV Power R. Combination Therapy With Sirolimus and Interleukin-2 Prevents Spontaneous and Recurrent Autoimmune Diabetes in NOD Mice. Diabetes (2002) 51:638–45. doi: 10.2337/diabetes.51.3.638 11872661

[126] Loschinski R Böttcher M Stoll A Bruns H Mackensen A Mougiakakos D. IL-21 Modulates Memory and Exhaustion Phenotype of T-Cells in a Fatty Acid Oxidation-Dependent Manner. Oncotarget (2018) 9:13125–38. doi: 10.18632/oncotarget.24442 PMC5862566 29568345

[127] Monteleone G Pallone F Macdonald TT. Interleukin-21 as a New Therapeutic Target for Immune-Mediated Diseases. Trends Pharmacol Sci (2009) 30:441–7. doi: 10.1016/j.tips.2009.05.006 19616319

[128] Ren HM Lukacher AE Rahman ZSM Olsen NJ. New Developments Implicating IL-21 in Autoimmune Disease. J Autoimmun (2021) 122:102689. doi: 10.1016/j.jaut.2021.102689 34224936 PMC8293794

[129] von Herrath M Bain SC Bode B Clausen JO Coppieters K Gaysina L. Anti-Interleukin-21 Antibody and Liraglutide for the Preservation of β-Cell Function in Adults With Recent-Onset Type 1 Diabetes: A Randomised, Double-Blind, Placebo-Controlled, Phase 2 Trial. Lancet Diabetes Endocrinol (2021) 9:212–24. doi: 10.1016/S2213-8587(21)00019-X 33662334

[130] Felton JL. Timing of Immunotherapy in Type 1 Diabetes: The Earlier, the Better? ImmunoHorizons (2021) 5:535–42. doi: 10.4049/immunohorizons.2000105 34261674

[131] Balyan R Gautam N Gascoigne NRJ. The Ups and Downs of Metabolism During the Lifespan of a T Cell. Int J Mol Sci (2020) 21:7972. doi: 10.3390/ijms21217972 PMC7663011 33120978

[132] Leone RD Powell JD. Metabolism of Immune Cells in Cancer. Nat Rev Cancer (2020) 20:516–31. doi: 10.1038/s41568-020-0273-y PMC8041116 32632251

[133] Patel CH Powell JD. Targeting T Cell Metabolism to Regulate T Cell Activation, Differentiation and Function in Disease. Curr Opin Immunol (2017) 46:82–8. doi: 10.1016/j.coi.2017.04.006 PMC5554728 28521236

